# Azole resistance mechanisms and population structure of the human pathogen *Aspergillus fumigatus* on retail plant products

**DOI:** 10.1128/aem.02056-23

**Published:** 2024-04-23

**Authors:** Caroline Wang, Natalie Miller, Douglas Vines, Paul M. Severns, Michelle Momany, Marin T. Brewer

**Affiliations:** 1 Fungal Biology Group, Plant Pathology Department, University of Georgia, Athens, Georgia, USA; 2 Fungal Biology Group, Plant Biology Department, University of Georgia, Athens, Georgia, USA; Anses, Maisons-Alfort Laboratory for Food Safety, Maisons-Alfort, France

**Keywords:** antifungal, aspergillosis, compost, triazole, mycoses, fungicide resistance

## Abstract

*Aspergillus fumigatus* is a ubiquitous saprotroph and human-pathogenic fungus that is life-threatening to the immunocompromised. Triazole-resistant *A. fumigatus* was found in patients without prior treatment with azoles, leading researchers to conclude that resistance had developed in agricultural environments where azoles are used against plant pathogens. Previous studies have documented azole-resistant *A. fumigatus* across agricultural environments, but few have looked at retail plant products. Our objectives were to determine if azole-resistant *A. fumigatus* is prevalent in retail plant products produced in the United States (U.S.), as well as to identify the resistance mechanism(s) and population genetic structure of these isolates. Five hundred twenty-five isolates were collected from retail plant products and screened for azole resistance. Twenty-four isolates collected from compost, soil, flower bulbs, and raw peanuts were pan-azole resistant. These isolates had the TR_34_/L98H, TR_46_/Y121F/T289A, G448S, and H147Y *cyp51A* alleles, all known to underly pan-azole resistance, as well as WT alleles, suggesting that non-cyp51A mechanisms contribute to pan-azole resistance in these isolates. Minimum spanning networks showed two lineages containing isolates with TR alleles or the F46Y/M172V/E427K allele, and discriminant analysis of principle components identified three primary clusters. This is consistent with previous studies detecting three clades of *A. fumigatus* and identifying pan-azole-resistant isolates with TR alleles in a single clade. We found pan-azole resistance in U.S. retail plant products, particularly compost and flower bulbs, which indicates a risk of exposure to these products for susceptible populations and that highly resistant isolates are likely distributed worldwide on these products.

**IMPORTANCE:**

*Aspergillus fumigatus* has recently been designated as a critical fungal pathogen by the World Health Organization. It is most deadly to people with compromised immune systems, and with the emergence of antifungal resistance to multiple azole drugs, this disease carries a nearly 100% fatality rate without treatment or if isolates are resistant to the drugs used to treat the disease. It is important to determine the relatedness and origins of resistant *A. fumigatus* isolates in the environment, including plant-based retail products, so that factors promoting the development and propagation of resistant isolates can be identified.

## INTRODUCTION

*Aspergillus fumigatus* is a saprotrophic fungus that is widely distributed across the world (1, 2). It decomposes plant matter; therefore, it is commonly located in areas where plant growth or decomposition is abundant, such as in crop production settings, flower gardens, and compost piles (2–5). *A. fumigatus* primarily reproduces asexually by means of small, lightweight spores that are easily made windborne resulting in their abundance in the air (6). The spores of *A. fumigatus* are regularly inhaled without causing health problems, but they can infect the lungs of immunocompromised individuals, those with chronic respiratory diseases, such as cystic fibrosis or tuberculosis, and patients with Corona Virus Disease 2019 (COVID-19) (6–8). *A. fumigatus* causes over 300,000 life-threatening infections each year with mortality rates of 30%–90% (9, 10). Aspergillosis is a spectrum of respiratory conditions caused by *A. fumigatus* and some other *Aspergillus* species that ranges in severity from asthma to invasive aspergillosis depending on the immune system function of the host (11). Most cases of aspergillosis are treated primarily with triazole drugs, which have similar molecular structures and the same modes of action (11, 12). Without treatment, or when infections are caused by resistant isolates, mortality for invasive aspergillosis approaches 100% (9, 10).

Triazoles (referred to from here on as azoles) prevent fungal growth by inhibiting the production of ergosterol, which is necessary to maintain structure and fluidity in the cell membranes of *A. fumigatus* (13). Specifically, azoles bind the 14-α-demethylase enzyme in the ergosterol biosynthetic pathway, which in *A. fumigatus* is encoded by *cyp51A*. However, in the late 1990s, isolates of *A. fumigatus* resistant to multiple azole drugs were found in aspergillosis patients with no prior exposure to azole drugs (14). The first and most common molecular mechanism of resistance identified in these isolates was a set of mutations in *cyp51A* combining a tandem repeat of 34 base pairs in the promoter region with a non-synonymous single nucleotide polymorphism in the coding region; this mutation is designated as TR_34_/L98H (14). Because invasive aspergillosis does not present with sporulation within the patient in most cases and person-to-person transmission of aspergillosis has not been documented, it was concluded that patients likely acquired isolates with the TR_34_/L98H allele from the environment rather than developing it as a result of azole therapy (15, 16). Additional mutations in *cyp51A* that convey similar resistance to multiple azole drugs arose thereafter, including TR_46_/Y121F/T289A, G448S, TR_34_/R65K/L98H, TR_53_, and others (17–20). Isolates with these alleles were found in patients with no prior exposure to azoles and showed a cross-resistance to agricultural azoles used to combat plant-pathogenic fungi, which indicated that the isolates had been exposed to azoles and developed resistance prior to inhalation by the patients (17, 21–23).

The widespread agricultural use of azoles is now considered the driving force for much clinical resistance in *A. fumigatus* (5, 17, 24–27) Though resistance has also been associated with long-term azole therapy in patients with chronic infections, at least two-thirds of patients with azole-resistant *A. fumigatus* infections have not previously undergone azole therapy (18, 22, 27, 28). During the last decade, Europe and Asia have seen an alarming increase in azole-resistant *A. fumigatus* in the clinic, and azole resistance is now present in patients and the environment on six continents (3, 25). Environmental isolates with azole resistance and nearly identical genotypes, including isolates from agricultural environments, have been documented in regions where resistant clinical strains have been found (22, 25, 26). In the United States (U.S.), azole-resistant *A. fumigatus* strains have been recently reported in patients (29–31) and the environment in agricultural settings (5, 32, 33).

The abundance of *A. fumigatus* in agricultural environments combined with the increased use of triazole fungicides (34) has led to intensified surveys for reservoirs and hot spots of azole resistance (3, 21, 35–40). Relatively few environmental surveys have been conducted in the U.S. to determine the prevalence of azole resistance in *A. fumigatus*; however, resistance has been found in a peanut debris waste pile, pecan debris, compost, and soil with plant debris where wheat, apple, peach, grape, herbs, tulip, and hemp are grown (5, 32, 33). Isolates of *A. fumigatus* have been recovered from numerous plant products, including pecans, pine nuts, walnuts, hazelnuts, peanuts, cereals (rice, barley, and wheat), soybeans, corn, baldi bread, cocoa, cheese, mango pickles, eggs, dried fish, meat, melon seeds, bananas, apples, apricots, oranges, lemons, peaches, kiwis, grapefruit juice, tea, dried onion, and tropical vegetables (41–46). However, azole resistance was not investigated in these studies. We hypothesize that azole-resistant *A. fumigatus* isolates are present on retail plant-based food products that have been harvested from settings where azole fungicides are frequently applied. Fresh market retail plant products, especially those with debris, such as nuts with shells and fruits with stems, could potentially support sporulating colonies of resistant *A. fumigatus* that would pose a threat to susceptible groups. Interestingly, azole-resistant *A. fumigatus* has been identified in retail tulip bulbs, retail seeds, retail soil, and retail compost in the Netherlands, the United Kingdom, and Japan (3, 27, 39, 47, 48). Nonetheless, no studies have investigated the possibility of azole-resistant *A. fumigatus* being spread through retail plant material in the U.S. In this study, we aimed to determine if azole-resistant *A. fumigatus* is present in retail products originating from agricultural production environments in the U.S. where azoles are commonly used, including fresh retail grapes, peanuts, pecans, almonds, and apples. We also investigated known hot spots of resistance that have not been studied in the U.S., including retail compost, soil, and flower bulbs. If azole resistance was detected, we aimed to determine the molecular mechanism of azole resistance in these isolates and explore how they are related to other isolates of *A. fumigatus* from agricultural and clinical environments.

## MATERIALS AND METHODS

### Collection and isolation of *A. fumigatus* from retail plant products

Eight product types were collected from retail stores from September 2019 through April 2021 and surveyed for azole-resistant *A. fumigatus*. Grapes, apples, almonds, pecans, peanuts, compost, soil, and flower bulbs (tulip, daffodil, *Gladiolus*, daylily, *Dahlia*, *Canna*, *Liatris*, *Caladium*, lily of the valley, *Clematis*, *Iris ensata*, and magnum elephant ears) were collected from eight retail grocery stores and nine garden centers in the Athens, Georgia area. The date of collection for fruits and nuts depended on their fresh market season in the U.S. Each product was collected by purchasing a pre-packaged bag. A wide variety of brands, product sizes, and retail stores were sampled. An effort was made to collect products that originated in the U.S.; however, some packages of soil and flower bulbs originated from Canada, China, Costa Rica, and the Netherlands, respectively.

Grapes were first sampled using amended sampling methods (39, 42), where parts of individual grapes including the skin and stems were swabbed or plated directly onto Sabouraud dextrose agar (SDA) amended with 50 µg/mL rose bengal dye, 50 µg/mL chloramphenicol, and 5 µg/mL gentamicin (aSDA) (5). However, this resulted in low yields of *A. fumigatus* and prevented the entire sample from being tested efficiently; therefore, the following method was devised, which was able to be used on grapes, peanuts, pecans, almonds, apples, and flower bulbs not packed in soil. The contents of the entire bag of the purchased product ranging from approximately 0.5 to 2 kg were placed into a sterile 30 × 38 cm polypropylene bag and rinsed thoroughly with 50 mL sterile 0.05% Tween-20 by massaging the bag to distribute the liquid over the plant products. All grapes contained stems and some peanuts and pecans were unshelled. All almonds were shelled. The liquid was collected into a 50 mL conical tube by cutting one corner of the bag. Almonds and pecans contained large quantities of broken shells, skin, and dust so the liquid was strained through sterile cheesecloth during collection. The tube was centrifuged at 3,000 × *g* for 5 min (49), and the supernatant was poured off. After observing that pellets from some products contained less *A. fumigatus* on the surface (grapes, pecans, and almonds), these samples were resuspended in 1 mL of 0.05% Tween-20, and 100 µL aliquots of the entire solution were plated and spread onto 5–15 Petri plates of aSDA so that we could obtain multiple isolates of *A. fumigatus*. Pellets from products observed with more abundant *A. fumigatus* (peanuts and flower bulbs not packed in soil) were resuspended in 2.5 mL 0.05% Tween-20 solution and distributed in 100 µL aliquots among 10 plates of aSDA, 10 plates of aSDA amended with 3 µg/mL itraconazole (ITC, Thermo Scientific Acros Organics, New Jersey, USA), and 10 plates of aSDA amended with 3 µg/mL tebuconazole (TEB, TCI America, Oregon, USA).

Flower bulbs packed in soil contained very abundant amounts of *A. fumigatus,* so they were processed differently. Bulbs were removed from the soil and placed into a separate bag, shaking off as much soil as possible first. The bulbs were rinsed with 0.05% Tween-20 as described above. The liquid collected into a 50 mL conical tube was immediately vortexed at maximum speed, and 3 mL was transferred to another tube, avoiding any soil that may have been collected in the liquid. The liquid was not centrifuged, unlike with other samples, because the amount of *A. fumigatus* present was so great that the sample did not need to be concentrated. One and a half milliliters of the suspension was spread plated in 100 µL aliquots onto five plates of aSDA, five plates of aSDA amended with 3 µg/mL ITC, and five plates of aSDA amended with 3 µg/mL TEB. The remaining liquid in the tube was diluted 1:2 with fresh 0.05% Tween-20, and 1.5 mL in 100 µL aliquots was spread plated among five plates of aSDA, five plates of aSDA amended with 3 µg/mL ITC, and five plates of aSDA amended with 3 µg/mL TEB. The additional dilution was plated in case the initial sample plating was not dilute enough to visualize individual colonies of *A. fumigatus*.

Compost and soil were sampled using an amended sampling method (5, 32). Briefly, 2 or 4 g of the material was suspended in 0.1 M sodium pyrophosphate. Only 2 g of soil and compost with lower densities were collected in order to accommodate the size limit of the 50 mL conical tubes, otherwise 4 g was collected. The suspensions were vortexed for 30 s and allowed to settle for 1 min. Two and a half milliliters of the supernatant was plated as described above for peanuts and flower bulbs not packed in soil.

All agar plates were incubated at 45°C for 2–4 days. Individual colonies that were confirmed as *A. fumigatus* based on morphology were quadrant-streaked onto SDA to obtain a single-spore culture. All colonies were preliminarily screened for azole resistance by quadrant-streaking onto SDA with 3 µg/mL of TEB and SDA with 3 µg/mL of ITC alongside known resistant and susceptible control isolates (5). Plates were incubated at 37°C for 2 days, after which resistance to TEB and ITC was preliminarily scored as resistant, intermediate, or susceptible based on a visual assessment of growth. Isolates scored as resistant had proficient growth in at least two quadrants of an aSDA plate; intermediate had spotty growth in one quadrant of an aSDA plate; and susceptible did not grow at all. Azole sensitivity was then quantified as described in the section below. For long-term storage, a single-spore colony was selected from SDA and streaked onto complete media (50) using a sterile cotton swab. For each isolate, conidia from complete media slants incubated for 2 days at 37°C were harvested, suspended in 15% glycerol in cryotubes, frozen in liquid nitrogen, and stored at −80°C.

### Azole-resistance phenotyping

A total of 111 of the isolates preliminarily scored as resistant and 19 of the isolates preliminarily scored as susceptible or intermediate in the screening were selected for azole-resistance phenotyping via minimum inhibitory concentration (MIC) assays to the fungicide TEB and clinical antifungals ITC, voriconazole (VOR; Thermo Scientific Acros Organics, New Jersey, USA), and posaconazole (POS; Apexbio Technology, Texas, USA). Isolates representing a variety of products and samples with elevated growth on azole-amended SDA were selected and assessed using the Clinical Laboratory Standard Institute broth microdilution method (51). Not all isolates were assayed at this stage since many had similar phenotypes from the same sample, such as the same bag of compost or flower bulbs. Some susceptible isolates were included for comparison. Briefly, conidia were harvested from 4-day-old complete media slants using 3 mL sterile 0.05% Tween-20. The spore suspensions were adjusted to 0.09–0.13 OD at 530 nm using a spectrophotometer, and 20 µL of suspension was added to 11 mL RPMI 1640 liquid medium (Thermo Scientific Gibco, California, USA). The solution was distributed in 100 µL aliquots among 96 wells in microtiter plates containing twofold serial dilutions of antifungals with the final concentrations ranging from 0.015625 to 16 µg/mL. The plates were incubated at 37°C for 48 h. The MIC of each isolate to each antifungal was determined visually by selecting the first well that had no fungal growth; the corresponding concentration of the antifungal in that well was the MIC. The accuracy of the MIC assays was checked using both susceptible and resistant *A. fumigatus* control isolates with known MIC values for TEB, ITC, VOR, and POS. The EUCAST breakpoints defined in February 2020 (52) were used to classify isolates as sensitive (S) or resistant (R): ITC S ≤ 1 µg/mL > R, VOR S ≤ 1 µg/mL > R, and POS S ≤ 0.125 µg/mL and 0.25 µg/mL > R. MIC values of 2 µg/mL for ITC and VOR and 0.25 µg/mL for POS are classified as areas of technical uncertainty meaning that treatment with these antifungals may be used for isolates with this resistance breakpoint under certain situations, but for this study they were considered resistant (52). A breakpoint cutoff for TEB was defined as >2 µg/mL according to previous studies (5). Antifungal resistance phenotypes were further classified into four categories: azole susceptible, TEB resistant, pan-azole resistant, and azole resistant. Isolates with no azole resistance were classified azole susceptible. Isolates with a TEB-resistant phenotype but no resistance to any medical azole were classified TEB resistant. Isolates that were either resistant to only one medical azole or one medical azole and TEB were classified as azole resistant. Finally, isolates with resistance to more than one medical azole were classified as pan-azole resistant.

### DNA extraction

Hyphae of 102 isolates, including 80 isolates screened by MIC assays and 22 susceptible isolates representing a variety of the sampled products, were grown from spores in a liquid complete medium for 16–20 h at 30°C in a 1 × *g* orbital shaker (5). Tissue was gathered by filtering through a 40 µm cell strainer and squeezing the residual liquid from the tissue using a sterile cotton swab. Approximately 100–200 mg of tissue was collected and set aside in 2-mL tubes. DNA extractions were performed according to the Qiagen DNeasy Plant Mini Kit protocol (Qiagen, Maryland, USA), with a few amendments (53). Briefly, buffer AP1 was warmed to 65°C for at least 10 min prior to use. Four hundred microliters of buffer AP1 and 4 µL of RNase A were added to each tube with fungal tissue and vortexed for at least 2 min until all tissue was suspended. Each sample was incubated at 65°C for 10 min, vortexing for 10 seconds three times throughout the incubation. One hundred thirty microliters of buffer P3 was added to each sample, vortexed, and then incubated at −20°C for 5 min. The samples were centrifuged for 5 min at 18,407 × *g* to pellet the remaining solids. The supernatant was pipetted into a QIAshredder mini spin column and centrifuged for 2 min at 18,407 × *g*. Five hundred microliters of the flow-through fraction was transferred to a new sterile 2 mL lock-lid tube, and 750 µL of buffer AW1 (1.5× volume) was added to the flow-through and immediately mixed by pipetting. Six hundred fifty microliters of this solution was pipetted into a DNeasy mini spin column and centrifuged for 1 min at 18,407 × *g*. The flow-through was discarded, and the previous step was repeated with the remaining solution. The DNeasy spin column was transferred into a fresh 2 mL tube, 500 µL of buffer AW2 was added, and the column was centrifuged for 1 min at 18,407 × *g*. The flow-through was discarded, and the previous step was repeated except the centrifugation lasted for 2 min rather than 1 min. The DNeasy spin column was transferred to a sterile 1.5 mL tube, and 50 µL of buffer AE was added to the membrane. The columns were incubated at room temperature for 5 min, then centrifuged at 18,407 × *g* for 1 min. This step was repeated once, and the DNA was stored at 4°C. The DNA concentration was quantified using NanoDrop One (Thermo Scientific, New Jersey, USA).

### *cyp51A* sequencing

Seventy-nine isolates, including 37 sensitive and 42 resistant isolates based on MIC assays, were selected for Sanger sequencing of the promoter and coding regions of *cyp51A*. These isolates were selected in order to obtain representatives of isolates with varying phenotypes from all sampled plant-based retail products. The selection of isolates was based primarily on elevated MIC values, but some susceptible isolates were included as well for comparison. Isolates with low TEB MIC values and isolates with similar MIC values from the same sample were not always included. PCR was performed using a mix of 12.5 µL OneTaq 2× Master Mix, 6.5 µL RNA-free sterile ddH_2_O, 2 µL each of previously designed forward primer 5′-CGGGCTGGAGATACTATGGCT-3′, and reverse primer 5′-GTATAATACACCTATTCCGATCACACC-3′ (5, 54). PCR cycling conditions were as follows: 98°C for 2 min followed by 30 cycles of 98°C for 15 s, 62°C for 15 s, and 72°C for 2.5 min, followed by a final extension at 72°C for 5 min (5). Sanger sequencing was performed by Genewiz (Genewiz by Azenta Life Sciences, Massachusetts, USA) using four primers: 5′-GCATTCTGAAACACGTGCGTAG-3′, 5′-GTCTCCTCGAAATGGTGCCG-3′, 5′-CGTTCCAAACTCACGGCTGA-3′, and 5′-GCGACGAACACTTCCCCAAT-3′ (5). Sequence alignment was performed using Geneious v2019.2 (Biomatters, Auckland, New Zealand). Briefly, all sequences were trimmed to remove low-quality base pairs with a base quality score <20 from the beginning and end of each sequence. The sequences were aligned for each isolate, and the consensus sequence was visually assessed. The promoter regions were aligned and compared with A1163 genomic sequence v43 from Ensembl (55). The coding sequences were translated and aligned to the *A. fumigatus* A1163 Cyp51A protein (GenBank accession EDP50065).

### STR*Af* genotyping

Single tandem repeats of *A. fumigatus* (STR*Af*) are microsatellite markers commonly used to assess genetic diversity and population genetic structure among isolates from different environmental origins, such as clinical or agricultural environments (56–59). Nine previously developed STR*Af* markers (STR*Af*2A, 2B, 2C, 3A, 3B, 3C, 4A, 4B, and 4C) were used to genotype 95 isolates collected in this study: 72 of which we had collected *cyp51A* sequence data and 23 others that were included to investigate isolates from all of the sampled plant-based retail products (57). Multiplex PCR was performed using a modified protocol for the Type-it Microsatellite PCR kit (Qiagen). Briefly, each of the three multiplex reactions (three loci per multiplex) contained 5 µL 2× Type-it Master Mix, 1 µL 10× primer mix (2 µM of each of the six multiplex primers), 1 µL DNA template, and RNase-free water. Thermal cycling conditions were as follows: 95°C for 5 min followed by 28 cycles of 95°C for 30 s, 57°C for 90 s, 72°C for 30 s, and a final elongation of 60°C for 30 min. Amplification of several PCR products from each multiplex was confirmed by electrophoresis on a 1% agarose gel with 1× TBE buffer. The PCR products were diluted 1:15 and then sent to the Cornell Institute of Bioinformatics (Ithaca, New York, USA) for the addition of the internal size standard Genescan-500 Liz and HiDi-formamide, followed by fragment analysis on an Applied Biosystems 3730 × 1 96-capillary DNA analyzer. The data were analyzed using the Microsatellite plugin in Geneious v.6 (Biomatters, Auckland, New Zealand) to identify the nine loci and the amplicon length (or allele) in each sample.

### Population genetic analyses

Multilocus genotypes used in the analyses were based on the STR*Af* data for the 95 isolates from this study and 80 clinical and environmental isolates from the U.S. from a previous study (5). There were 28 isolates from clinical settings, and environmental isolates came from agricultural compost (4) and soil with plant debris where apple (2), watermelon (7), strawberry (4), pecan (13), peanut (16), and grape (5) were growing. The laboratory reference used in the previous study (5), Af293, was included as a control for comparison. Isolates from a variety of substrates were chosen to obtain a representative sample, but substrates associated with the retail products sampled in this study were prioritized (e.g., soil used to grow grape, apple, peanut, and peanut debris).

To estimate the genetic relatedness among isolates, minimum spanning networks using Bruvo’s genetic distance model (60) and Nei’s 1978 distance (61, 62) were constructed with the Poppr package in R (63). Bruvo’s genetic distance is often useful for analyses based on microsatellite markers (60) and assumes a stepwise mutation model that may not be entirely accurate for an organism like *A. fumigatus,* which is genotypically diverse and known to sexually reproduce (64). Moreover, each STR*Af* locus contains 11–37 alleles that vary in repeat number, so they may not be evolving in a stepwise manner (57). Therefore, we used Nei’s genetic distance, as well, to incorporate an infinite alleles model. Population genetic structure was analyzed using discriminate analysis of principal components (DAPC) in R (65) to identify if populations clustered based on environmental setting, substrate of origin, *cyp51A* genotype, geographic sampling location, or another factor. Clusters were determined using K-means clustering of principal components.

## RESULTS

### Isolate collection and phenotyping

For this study, a total of 525 *A*. *fumigatus* isolates were obtained from grapes, peanuts, pecans, almonds, apples, compost, soil, and flower bulbs (Table 1). Multiple samples yielded no *A. fumigatus* isolates confirming that the sterile bags used to wash samples were not likely the source of any isolates. The greatest number of *A. fumigatus* isolates (90% of our collection) was obtained from peanuts, flower bulbs, soil, and compost. Flower bulbs with high quantities of *A. fumigatus* included tulip, *Dahlia*, and lily of the valley, packed in both soil and alone, and originating from the Netherlands (Table S1). Compost made with cow manure had greater quantities of *A. fumigatus* (Table S1). Few isolates were recovered from apple, grape, and almond samples. Based on the initial screening on azole-amended media, four isolates from pecan, soil, and compost were preliminarily scored resistant to TEB, 105 from peanuts, compost, flower bulbs, and soil were preliminarily scored resistant to ITC, and 81 isolates from compost, flower bulbs, and soil were preliminarily scored resistant to both TEB and ITC (Table 1). Most of the isolates preliminarily scored as resistant were found in soil, compost made from manure, and bulbs of daffodil, *Dahlia*, *Gladiolus*, and tulip (Table S1).

**TABLE 1:** *Aspergillus fumigatus* isolates obtained from retail plant products in the United States

Plant-based retail product	No. isolates	Growth on aSDA			Selected for MIC*^d^*	No. resistant isolates				Azole-resistance phenotype^*g*^			
		TEB^*a*^	ITC^*b*^	TEB and ITC^*c*^		TEB^*e*^	ITC*^f^*	VOR	POS	Sens.	Teb.	Med.	Pan.
Almond	2	0	0	0	2	2	1	0	0	0	1	1	0
Apple	2	0	0	0	0	0	0	0	0	0	0	0	0
Grape	35	0	0	0	8	0	0	0	0	8	0	0	0
Peanut	147	0	33	0	33	4	2	1	1	29	2	1	1
Pecan	12	1	0	0	6	4	1	0	0	2	3	1	0
Flower bulbs	109	0	9	42	20	15	9	15	13	5	0	2	13
Compost	133	2	43	33	44	25	10	9	9	18	15	2	9
Soil	85	1	20	6	17	5	1	1	1	12	4	0	1
Total	525	4	105	81	130	55	24	26	24	74	25	7	24

^*a*^ SDA amended with 3 µg/mL tebuconazole.

^*b*^ SDA amended with 3 µg/mL itraconazole.

^*c*^ Number of isolates that grew both on SDA amended with 3 µg/mL tebuconazole and SDA amended with 3 µg/mL itraconazole.

^*d*^ Isolates selected for MIC assays.

^*e*^ Resistance set at >2 µg/mL based on prior studies (5).

^*f*^ Resistance breakpoints for ITC, VOR, and POS based on EUCAST 2020 breakpoints: ITC and VOR > 1 µg/mL, POS > 0.25 µg/mL.

^*g*^ Sens., sensitive to all four azoles tested; Teb., resistant only to TEB fungicide; Med., resistant to one medical azole; and Pan., pan-azole-resistant, resistant to more than one medical azole.

Resistance to TEB, ITC, VOR, and POS was quantified by MIC assays for 130 isolates (Table 1). Isolates were chosen based on preliminary resistance screening data and product of origin targeting mostly resistant but also including some sensitive isolates from a variety of products. In total, 42.3% (*n* = 55) were resistant to TEB, 18.5% (*n* = 24) were resistant to ITC, 20.0% (*n* = 26) were resistant to VOR, and 18.5% (*n* = 24) were resistant to POS (Table 1). All isolates resistant to medical azoles, with one exception, were also resistant to TEB. Isolates resistant to at least one of the four tested azoles were found in all products except apple and grape (Table 1). Overall, 57% (*n* = 74) of the isolates assayed by MIC were azole sensitive, 19% (*n* = 25) were classified as TEB resistant, 5% (*n* = 7) were classified as azole resistant, and 19% (*n* = 24) were classified as pan-azole resistant (Table 1). Seven isolates from almonds, flower bulbs, compost, peanuts, and pecans were resistant to one medical azole with or without TEB resistance and classified as azole resistant (Tables 1 and 2). One isolate each from a peanut, pecan, and almond sample was inhibited at concentrations of 4 µg/mL TEB and 2 µg/mL ITC (Table 2). Two of the isolates from flower bulbs classified as azole resistant had elevated MICs to TEB and VOR (Tables 1 and 2). One isolate from compost had MIC values of 8 µg/mL for TEB and 2 µg/mL for ITC, and the other compost isolate had an MIC of 0.5 µg/mL for POS (Table 2). There were 24 total isolates classified as pan-azole resistant (Tables 1 and 2). Of these, 9 were from compost, 1 was from soil, 13 were from flower bulbs, and 1 was from an unshelled raw peanut (Tables 1 and 2; Table S1). The pan-azole-resistant isolates from compost were mainly from compost consisting of cow and hen manure (Table S1). Pan-azole-resistant isolates from flower bulbs were from daffodil, *Dahlia*, daylily, *Gladiolus*, and tulip (Table S1).

**TABLE 2:** *Aspergillus fumigatus* isolates with azole and pan-azole resistance

Product	Isolate ID	MIC value (µg/mL)				*cyp51A* phenotype^*c*^	*cyp51A* genotype^*d*^
		TEB^*a*^	ITC^*b*^	VOR	POS		
Almond	183	4	2	0.5	0.25	Azole resistant	F46Y/M172V/N248T/D255E/E427K
Flower bulb	491	17	0.5	17	0.25	Azole resistant	TR_46_/Y121F/T289A/G448S
Compost	221	2	1	1	0.5	Azole resistant	F46Y/M172V/E427K
Flower bulb	517	8	1	2	0.25	Azole resistant	WT
Peanut	196	4	2	1	0.25	Azole resistant	WT
Compost	212	8	2	1	0.25	Azole resistant	Not sequenced
Pecan	188	4	2	1	0.25	Azole resistant	Not sequenced
Soil	385	17	2	17	0.5	Pan-azole resistant	TR_46_/Y121F/T289A
Compost	398	17	2	17	0.5	Pan-azole resistant	TR_46_/Y121F/T289A
Compost	399	17	2	17	1	Pan-azole resistant	TR_46_/Y121F/T289A
Flower bulb	419	17	8	17	4	Pan-azole resistant	TR_46_/Y121F/T289A/G448S
Flower bulb	432	17	4	17	2	Pan-azole resistant	TR_46_/Y121F/T289A/G448S
Flower bulb	449	17	1	17	0.5	Pan-azole resistant	TR_46_/Y121F/T289A
Flower bulb	483	17	1	17	1	Pan-azole resistant	TR_46_/Y121F/M172I/T289A
Flower bulb	487	17	1	17	1	Pan-azole resistant	TR_46_/Y121F/T289A/G448S
Flower bulb	496	17	2	17	1	Pan-azole resistant	TR_46_/Y121F/T289A/G448S
Flower bulb	501	17	1	17	1	Pan-azole resistant	TR_46_/Y121F/T289A/G448S
Flower bulb	433	17	17	8	2	Pan-azole resistant	TR_34_/L98H
Flower bulb	434	17	17	8	1	Pan-azole resistant	TR_34_/L98H
Flower bulb	438	17	17	8	1	Pan-azole resistant	TR_34_/L98H
Flower bulb	439	17	17	8	1	Pan-azole resistant	TR_34_/L98H
Flower bulb	506	17	17	8	1	Pan-azole resistant	TR_34_/L98H
Flower bulb	448	17	4	4	0.5	Pan-azole resistant	TR_34_/L98H/D255E
Peanut	130	16	16	8	2	Pan-azole resistant	TR_34_/L98H
Compost	245	16	4	4	0.5	Pan-azole resistant	WT
Compost	247	16	4	4	0.5	Pan-azole resistant	WT
Compost	257	16	4	4	1	Pan-azole resistant	WT
Compost	253	16	2	2	0.5	Pan-azole resistant	WT
Compost	263	16	2	2	0.125	Pan-azole resistant	WT
Compost	237	8	2	4	0.5	Pan-azole resistant	H147Y
Compost	206	16	4	2	0.5	Pan-azole resistant	Not sequenced

^*a*^ Resistance set at >2 µg/mL based on prior studies (5).

^*b*^ Resistance breakpoints for ITC, VOR, and POS based on EUCAST 2020 breakpoints: ITC and VOR >1 µg/mL, POS > 0.25 µg/mL.

^*c*^ Azole resistant, resistant to one medical azole; pan-azole resistant, resistant to more than one medical azole.

^*d*^ Sequences aligned to A1163 Cyp51A.

### *cyp51A* genotyping

**Fig 1:**
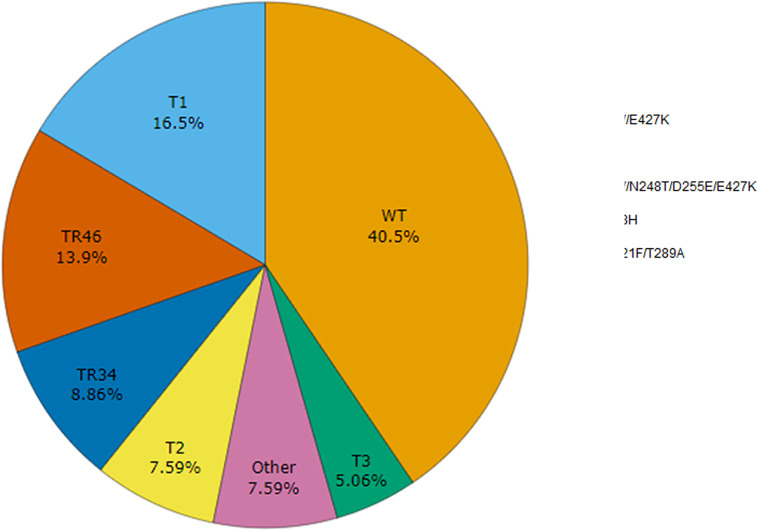
*cyp51A* genotypes of *Aspergillus fumigatus* from plant-based retail products. TR34 represents all isolates with a 34-base pair tandem repeat in the *cyp51A* promoter. TR46 represents all isolates with a 46-base pair tandem repeat in the *cyp51A* promoter. Other variants were only detected once. WT alleles are the same as isolate A1163. A total of 79 isolates were sequenced.

To investigate the molecular mechanisms of azole resistance, 79 isolates were selected for *cyp51A* sequencing of the promoter and coding region. Of the isolates selected, 23 were classified as pan-azole resistant, 37 were sensitive, 14 were TEB resistant, and 5 were resistant to one medical azole (Fig. 1). The alleles are notated based on comparison with the A1163 Cyp51A amino acid sequence. We detected 19 distinct *cyp51A* genotypes that were assigned to seven categories with one of the categories (“Other”) representing six variants only detected once, and two of the categories representing all variants of both the TR_34_ and TR_46_ alleles (Fig. 1). Isolates with a 34- or 46-base pair tandem repeat in the *cyp51A* promoter were found in 9% (*n* = 7) and 14% (*n* = 11) of the sequenced isolates, respectively (Fig. 1). Of these, six isolates had the TR_34_/L98H allele, one isolate had the TR_34_/L98H/D255E allele, four isolates had the TR_46_/Y121F/T289A allele, one isolate had the TR_46_/Y121F/M172I/T289A allele, and six isolates had the TR_46_/Y121F/T289A/G448S allele. Here, these isolates will be referred to as TR_34_/L98H and TR_46_/Y121F/T289A when they are being grouped together by their tandem repeat alleles. The F46Y/M172V/E427K allele was found in 16.5% (*n* = 13) of isolates, and I242V was found in 7.6% (*n* = 6) of isolates (Fig. 1). The WT allele matching A1163 was the most common among the isolates selected for *cyp51A* sequencing and was detected in 40.5% (*n* = 32) of isolates. The F46Y/M172V/N248T/D255E/E427K allele matching Af293 (55) and other alleles were found in 5% (*n* = 4) and 7.6% (*n* = 6) of isolates, respectively. The “other alleles” category included six distinct variants each found once in this study: A9T, F46Y/E427K, F46Y, F46Y/M172V/N248T/D255E/W415G/E427K, H147Y, and M172I.

**Fig 2:**
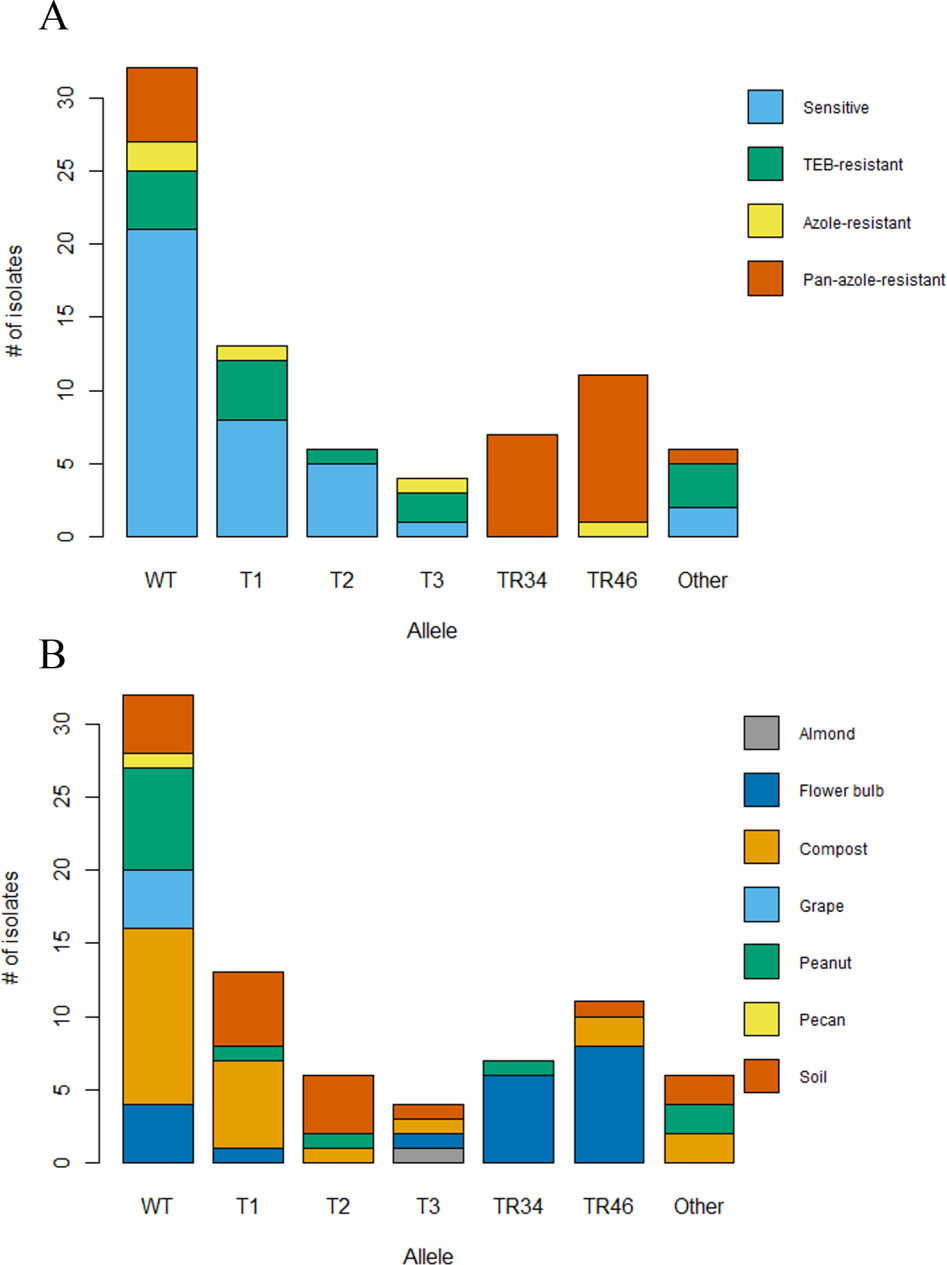
Proportion of *Aspergillus fumigatus* isolates with each *cyp51A* genotype by (A) azole-resistance phenotype and (B) retail plant-based product of origin. Pan-azole resistant denotes resistance to more than one medical azole (ITC, VOR, and POS) and the fungicide TEB. TEB resistant denotes resistance only to tebuconazole. Sensitive denotes susceptible to all four tested azoles. Azole resistant denotes resistance to only one medical azole (and possibly TEB). TR34 indicates the TR_34_/L98H allele. TR46 indicates the TR_46_/Y121F/T289A allele. T1 indicates the F46Y/M172V/E427K allele. T2 indicates the I242V allele. T3 indicates the F46Y/M172V/N248T/D255E/E427K allele. WT indicates the same sequence as *A. fumigatus* A1163. “Other” indicates a variant detected only once.

All isolates with the TR_34_/L98H allele showed a pan-azole-resistant phenotype, while all but one of the isolates with the TR_46_/Y121F/T289A allele showed pan-azole-resistant phenotypes (Fig. 2A). However, this isolate was classified as azole resistant since it was only resistant to VOR and TEB with MIC values of >16 µg/mL for each. Isolates with the F46Y/M172V/E427K and I242V alleles were sensitive to medical azoles, but some were resistant to TEB. One isolate with the F46Y/M172V/E427K allele was resistant to the medical azole POS (Fig. 2A). Isolates with the WT allele matching A1163 were primarily azole sensitive, but some were resistant to TEB (*n* = 4) or one medical azole (*n* = 2), and five were pan-azole resistant (Fig. 2A). Isolates with the F46Y/M172V/N248T/D255E/E427K allele matching Af293 had varying levels of resistance to azoles. Most isolates in the “other” alleles category were resistant to TEB or were completely susceptible, while one was resistant to ITC (Fig. 2A). Aside from isolates with the tandem repeat alleles, pan-azole resistance was found in isolates with WT and H147Y alleles (Fig. 2A). The majority of isolates with the TR_34_/L98H allele, except one from a raw peanut, and all isolates with the TR_46_/Y121F/T289A allele originated from lawn and garden products, which included compost, soil, or flower bulbs (Fig. 2B). Fewer *A. fumigatus* isolates were obtained and sequenced for *cyp51A* from food products (almond, grape, peanut, and pecan) than lawn and garden products, so food products were the minority of every category, but most isolates from food products had the WT allele (Fig. 2B).

### Population genetic structure

**Fig 3:**
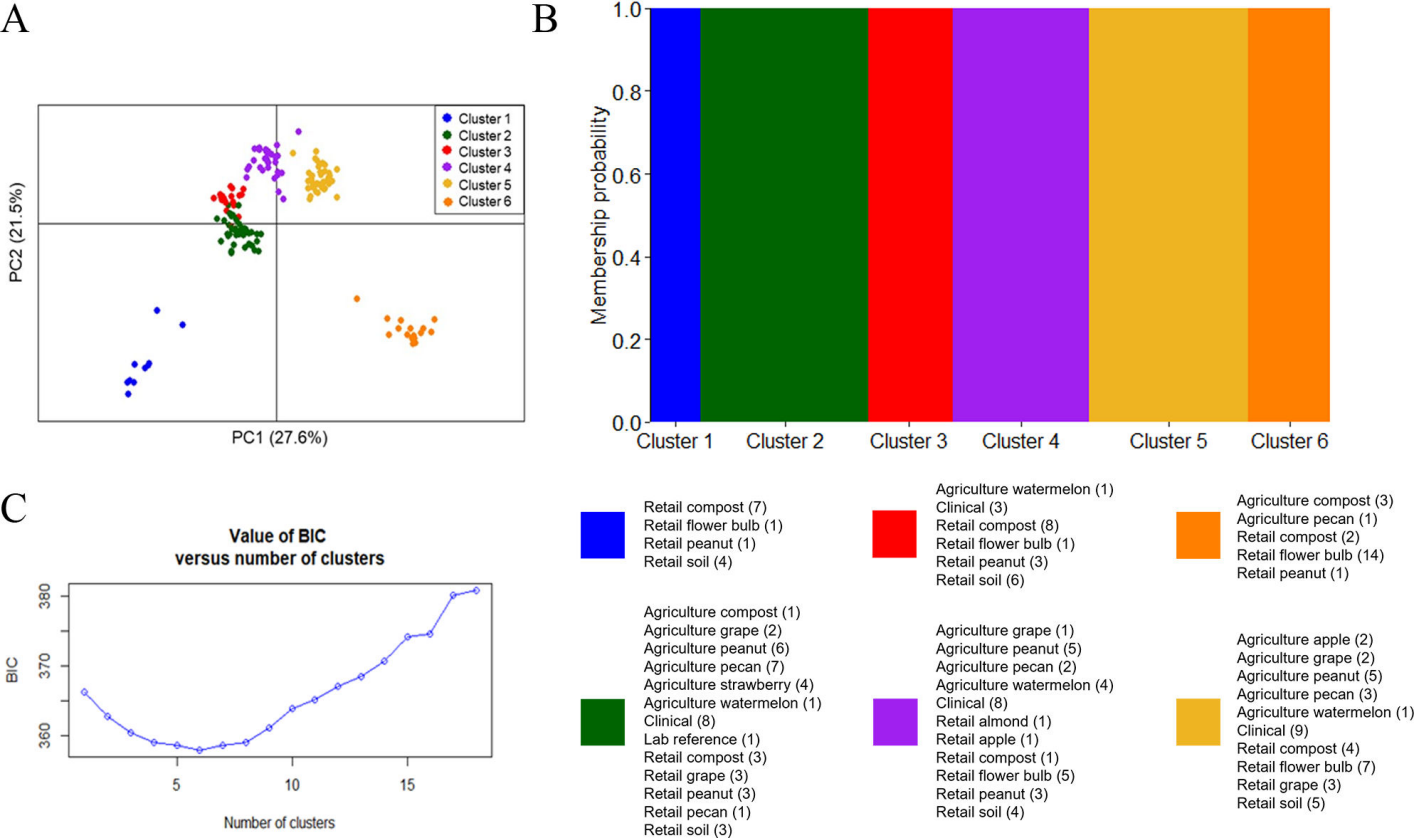
Clustering of *A. fumigatus* isolates from the U.S. by discriminant analysis of principal components based on genotyping of nine STR*Af* loci. (**A**) DAPC scatter plot of the first two principal components. Each dot represents an isolate, and the color indicates the cluster composition. (**B**) The Bayesian information criterion (BIC) for each *K* number of clusters. (**C**) Histogram of membership probability of 185 *A*. *fumigatus* isolates based on DAPC. The color indicates the cluster composition. The sampling environments or substrates of isolates in each cluster are listed below the histogram, and the number in parentheses indicates the number of isolates in that cluster from that substrate or setting.

To compare the genetic relationships of isolates from retail plant products with those from agricultural environments where the products are produced and those from patients, we conducted population genetic analyses on 95 isolates obtained for this study and 51 environmental and 29 clinical patient isolates from the U.S. that were previously collected (5). Population genetic structure was characterized by DAPC (Fig. 3A) with an optimum of *K* = 6 clusters identified. All isolates had 100% assignment probability to a single cluster (Fig. 3B). Principle component (PC) 1 and PC2 explained 27.6% and 21.5% of the variation, respectively (Fig. 3A). Each cluster was composed of a variety of isolates that originated from different environments. To distinguish samples from different environments, the isolates collected from this study all have retail in their name (e.g., retail grape). Samples with “agriculture” in their name were either compost from an agricultural setting (agriculture compost) or soil and/or plant debris in an agricultural setting where the crop was grown or processed (e.g., agriculture grape). Cluster 1 contained isolates exclusively from this study: retail flower bulb, retail compost, retail peanut, and retail soil (Fig. 3B). Cluster 2 contained isolates from the most diverse substrates and environments: agriculture compost, clinical, retail compost, retail grape, agriculture grape, lab reference strain Af293, retail peanut, agriculture peanut, retail pecan, agriculture pecan, retail soil, agriculture strawberry, and agriculture watermelon (Fig. 3B). Cluster 3 contained isolates from retail flower bulb, clinical, retail compost, retail peanut, retail soil, and agriculture watermelon (Fig. 3B). Cluster 4 contained isolates from retail almond, retail apple, retail flower bulb, clinical, retail compost, agriculture grape, retail peanut, agriculture peanut, agriculture pecan, retail soil, and agriculture watermelon (Fig. 3B). Cluster 5 contained isolates from agriculture apple, retail flower bulb, clinical, retail compost, retail grape, and agriculture grape (Fig. 3B). Finally, Cluster 6 contained isolates from agriculture compost, retail compost, retail flower bulb, retail peanut, and agriculture pecan (Fig. 3B).

**Fig 4:**
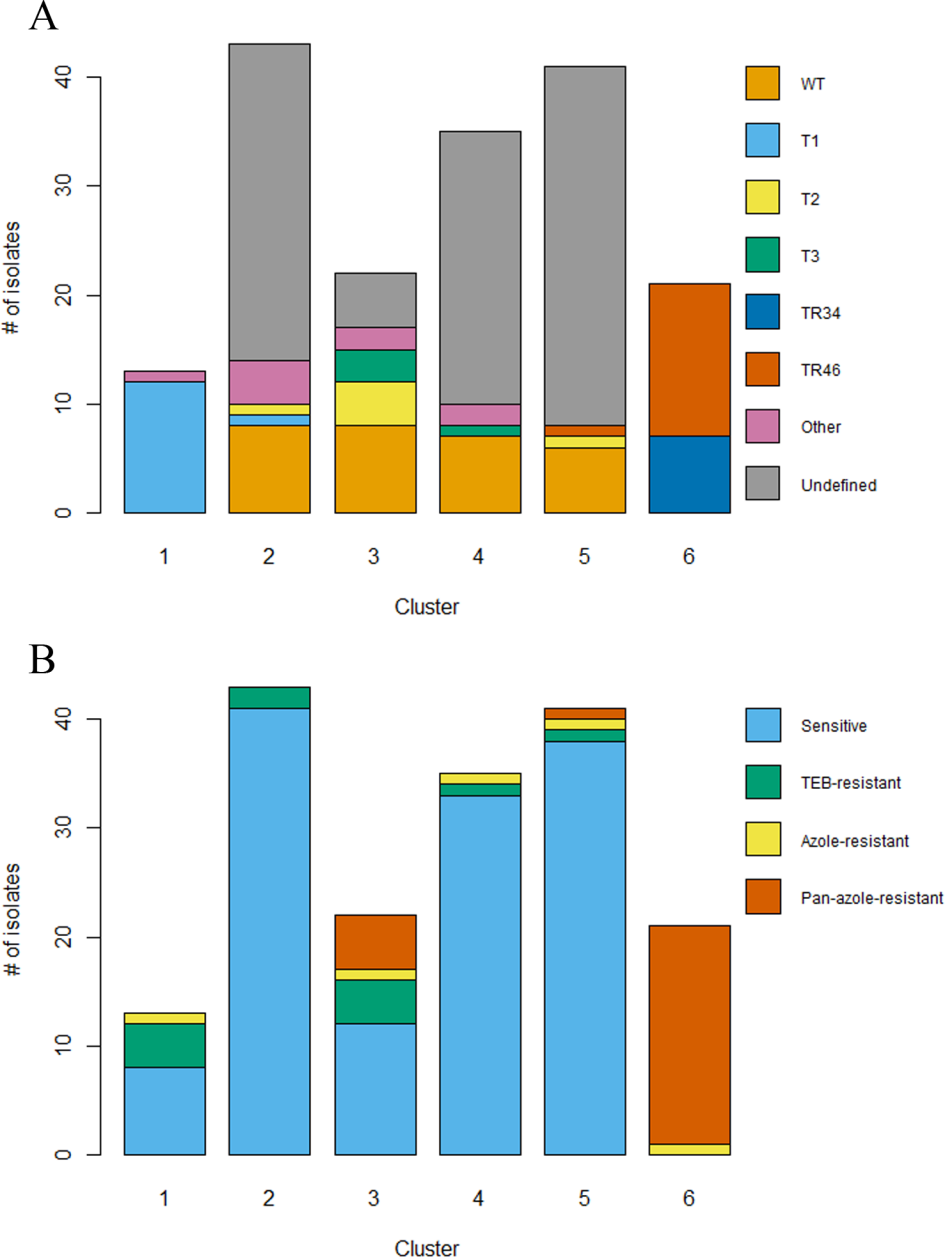
Proportion of *Aspergillus fumigatus* isolates in each genetic cluster by (A) *cyp51A* genotype and (B) azole-resistance phenotype. TR34 indicates the TR_34_/L98H allele. TR46 indicates the TR_46_/Y121F/T289A allele. T1 indicates the F46Y/M172V/E427K allele. T2 indicates the I242V. T3 indicates the F46Y/M172V/N248T/D255E/E427K allele. WT indicates the same sequence as *A. fumigatus* A1163. Other indicates a variant detected only once.

**Fig 5:**
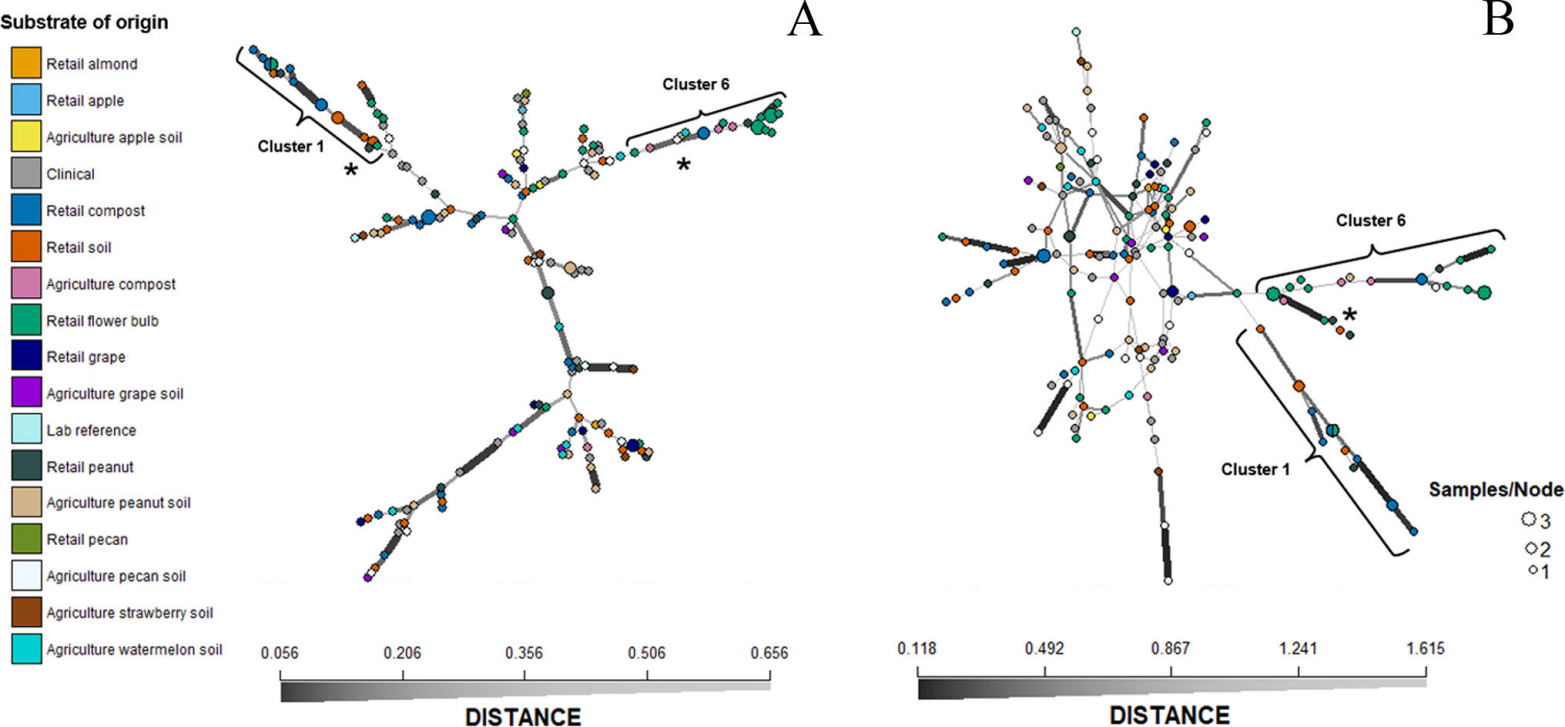
Minimum spanning networks of *Aspergillus fumigatus* isolates from environmental retail and clinical settings based on (A) Bruvo’s genetic distance and (B) Nei’s genetic distance. Each circle represents an STR*Af* genotype. The size of each circle indicates the proportion of isolates with that genotype with most genotypes represented by a single isolate. The color of the circles indicates the original substrate. The line thickness represents the genetic distance between each genotype. Asterisks represent one or more isolates that are part of a separate cluster identified by DAPC but are spatially grouped with either Cluster 1 or 6.

Many population genetic studies have detected either two or 3 clusters, clades, or groups within *A. fumigatus* (26, 31, 33, 66–69). Moreover, we saw close association among the isolates in Clusters 2, 3, 4, and 5 based on PC1 and PC2 (Fig. 3A), so we will discuss these together for easier comparison (Fig. 4). Next, we investigated if the *cyp51A* alleles of isolates showed patterns among the clusters. Clusters 1 and 2–5 comprised mostly of isolates with a non-TR genotype (Fig. 4A). Cluster 1 primarily contained isolates with the F46Y/M172V/E427K allele, with one isolate having a F46Y/E427K allele (Fig. 4A). This cluster also has a variety of resistance phenotypes, with the majority having a susceptible or TEB-resistant allele (Fig. 4B). However, one isolate with the F46Y/M172V/E427K allele was resistant to POS. Not all isolates in Clusters 2–5 were sequenced, so corresponding *cyp51A* data (Fig. 4A) are only available for 49 out of the 141 isolates from this cluster based on STR*Af* genotyping and DAPC (Fig. 3). Clusters 2–5 include isolates displaying a variety of azole-resistance phenotypes (Fig. 4B). There is one isolate with the TR_46_/Y121F/T289A allele that falls into this cluster (Cluster 5), while the other isolates have non-TR alleles (Fig. 4A). Most of the isolates in this cluster have an azole-susceptible phenotype; however, the isolate with the TR_46_/Y121F/T289A allele has a pan-azole-resistant phenotype (Fig. 5B). Cluster 6 comprises isolates with the TR_34_/L98H and TR_46_/Y121F/T289A *cyp51A* alleles and is almost entirely pan-azole-resistant isolates (Fig. 4). A single TR_46_/Y121F/T289A isolate in this cluster displayed resistance to only one medical azole. Further investigation of the alleles shows five distinct variants in this cluster, incorporating many of the non-synonymous substitutions observed in the other clusters (Table S1). However, the most common alleles found in Cluster 6 were the TR_34_/L98H allele with no additional substitutions found in 28% (*n* = 6) of isolates, and the TR_46_/Y121F T289A allele with no additional substitutions found in 19% (*n* = 4) of isolates.

To further explore genetic relationships among genotypes, minimum spanning networks were constructed based on Bruvo’s genetic distance and Nei’s genetic distance (Fig. 5). There is a great amount of genetic diversity present in the U.S. *A. fumigatus* population; nearly every isolate had a unique genotype. Only two isolates originating from different products (retail compost and retail flower bulbs) shared the same STR*Af* genotype. Interestingly, they also shared the same *cyp51A* allele (F46Y/M172V/E427K) and resistance phenotype (sensitive), indicating they are likely clones. Overall, there was no apparent genetic similarity based on the original setting for retail, clinical, or environmental isolates. However, there were two distinct branches in both networks that were associated with the F46Y/M172V/E427K allele of *cyp51A* and Cluster 1 or a tandem repeat in the promoter of *cyp51A* and Cluster 6 (Fig. 5), respectively. Genotypes in the Cluster 1 lineage were connected by thick lines indicating high genetic similarity despite being from different substrates, including retail compost, retail soil, retail flower bulbs, and retail peanut (Fig. 5). In both networks, there were several isolates not belonging to DAPC Cluster 1 or 6 that were on the same network branches as isolates from Clusters 1 or 6 (Fig. 5). The isolate that did not align with the DAPC Cluster 1 in the Bruvo’s genetic distance model was from a Cluster 6 flower bulb, and the isolates that did not align with DAPC Cluster 6 were from agriculture peanut and retail soil in Cluster 2. In Nei’s infinite alleles model, there were two isolates from retail peanuts and one from retail soil that aligned with Cluster 6 despite them being from Cluster 2. The network constructed with Nei’s genetic distance (Fig. 5B) displayed high genetic distance and branching patterns, suggesting past recombination within and between nodes of the network center; however, the evidence of a potential trend was not as well supported in the network constructed with Bruvo’s distance (Fig. 5A).

## DISCUSSION

To gain a better understanding of how widespread azole-resistant *A. fumigatus* is in the environment, we conducted a survey of azole-resistant *A. fumigatus* from retail, plant-based food items produced in the U.S. where azole fungicides are widely applied in their production. Additionally, we sampled retail lawn and garden products, including compost, flower bulbs, and soil, because these substrates are known to contain high levels of azole-resistant *A. fumigatus* (27, 35) and could be a potential source of both exposure to and dispersal of highly resistant isolates. Grapes, peanuts, pecans, almonds, apples, flower bulbs, soil, and compost were identified as products of interest because they are heavily managed with frequent azole fungicide sprays to combat plant-pathogenic fungi (70), or they are retail products of known azole-resistant *A. fumigatus* hotspots in other regions of the world (3, 27, 39). The food items surveyed were produced in the U.S., while the origin of the lawn and garden products (flower bulbs, soil, and compost) was either unknown, the U.S., the Netherlands, Costa Rica, China, or Canada (Table S1). We isolated 525 *A*. *fumigatus* from these products (Table 1; Table S1); however, most were recovered from peanut and lawn and garden products with few coming from other food items. Isolates from peanuts were only obtained from raw peanuts in their shells, not from any peanuts with either their shells removed or treated with heat (roasted or boiled) or salt.

Azole resistance screening of these isolates on media amended with 3 µg/mL TEB or 3 µg/mL ITC revealed that 4 and 105 of the isolates, respectively, grew on these concentrations of the antifungals, and 81 grew on both (Table 1), but resistance to multiple azoles had to be confirmed and further quantified by MIC assays since azole-sensitive isolates can sometimes grow at these lower concentrations in solid medium (5). A selection of the preliminarily screened isolates and some azole-susceptible isolates for comparison were selected for resistance phenotyping using MIC assays with TEB, ITC, VOR, and POS. In total, almost half of the 130 isolates evaluated by MIC (42.3%) were resistant to TEB (Table 1), suggesting that prior exposure to TEB had been selected for resistance or that there was sampling bias in our preliminary screen for resistant isolates. Nearly 20% of the isolates tested by MIC were resistant to the medical azoles ITC, VOR, or POS, and of these, 24 were pan-azole resistant (Tables 1 and 2).

Isolates from the azole-resistant (resistance to one medical-use azole) and especially the pan-azole-resistant phenotypes pose the greatest threat to public health. Compost, flower bulbs, and soil were the products of origin for most of the azole-resistant and pan-azole-resistant isolates aside from one isolate from peanut that was pan-azole-resistant; however, one isolate each from the retail nut products, including peanut, pecan, and almond, was resistant to ITC with an MIC of 2 µg/mL. All pan-azole-resistant isolates from flower bulbs were found on daffodil, *Gladiolus*, daylily, *Dahlia*, and tulip from the Netherlands or an unlisted location. The two azole-resistant isolates from flower bulbs were found on a tulip from an unknown location and the canna lily listed on the package as “produced in the U.S./Costa Rica.” All isolates with a pan-azole-resistant or azole-resistant phenotype, except one isolate from compost, were also resistant to TEB (Tables 1 and 2). This finding, along with the high overall proportion of TEB-resistant isolates, suggests that resistance to TEB, an azole widely used in the environment (34), may either facilitate or be associated with higher levels of azole resistance, including medical-use azoles. This finding is consistent with other studies reporting that both the TR_34_/L98H and the TR_46_/Y121F/T289A alleles may be selected for by the presence of tebuconazole (22, 71).

We detected 24 pan-azole-resistant isolates among the 525 isolates collected from plant-based retail products (Tables 1 and 2). The pan-azole-resistant isolates generally had three pan-azole-resistance phenotype profiles: (i) very high resistance to TEB and VOR (MIC > 16 µg/mL), and less resistance to ITC and POS (MIC 0.5–8 µg/mL), (ii) very high resistance to TEB and ITC (≥16 µg/mL, except in one isolate with an ITC value of 4 µg/mL), and less resistance to VOR and POS (0.5–8 µg/mL), and (iii) high resistance to TEB (8 µg/mL or greater), and less resistance to ITC, VOR, and POS (0.125–4 µg/mL). Interestingly, these phenotype profiles corresponded with *cyp51A* genotypes. Isolates with phenotype profile 1 had TR_46_/Y121F/T289A alleles, isolates with phenotype profile 2 had TR_34_/L98H alleles, and isolates with phenotype profile 3 had non-TR alleles, mainly the WT allele matching A1163 and one isolate with a H147Y substitution (Table 2). It is important to note that many sensitive isolates also had the WT allele, so it is clear that a non-cyp51A mechanism underlies pan-azole resistance in these isolates. Other studies have presented similar relationships among these phenotypes and genotypes (72, 73). Aside from the TR_34_/L98H and TR_46_/Y121F/T289A alleles, and the G448S substitution found among isolates also containing TR_46_/Y121F/T289, the H147Y variant is the only other mutation identified in our study known to underly azole resistance (74). H147Y has been found to cause elevated MIC values to ITC, VOR, and POS, so this is likely the molecular resistance mechanism responsible for the pan-azole resistance in this isolate (74). It is interesting that the pan-azole-resistant isolates with the lowest resistance levels to ITC, VOR, and POS (Table 2) have non-TR, WT alleles shared with azole-susceptible isolates. It is possible that pan-azole resistance is being induced by exposure to agricultural azoles in these isolates, which is supported by the high TEB MIC values respective to ITC, VOR, and POS in these isolates. The resistance mechanism in these isolates is not *cyp51A*-based, and another mechanism is responsible for the resistance. It is estimated that 50% of azole resistance in *A. fumigatus* is non-*cyp51A* based on mutations in genes such as *cdr1B, hapE,* and *hmg1* (75–77). Additional studies are needed to elucidate the mechanism of resistance of these isolates.

The isolates without tandem repeats in the *cyp51A* promoter region had a variety of non-synonymous substitutions in their *cyp51A* genes. Isolates with some of these substitutions have been associated with azole resistance, including F46Y, H147Y, M172V, N248T, D255E, E427K, G448S, 1242V, and A9T (67, 74, 78–80). However, only H147Y and G448S have been determined to cause azole resistance (67, 74, 81). The F46Y, M172V, N248T, D255E, and E427K substitutions have also been shown to be both present and absent in various susceptible reference strains, such as Af293, A1163, and AF338659 (82) and, therefore, do not likely underly azole resistance. Further studies are required to determine how various cyp51A substitutions affect azole resistance.

To understand how isolates from commercial products are related to isolates from the environments where they are produced in the U.S. and to clinical isolates in this region, we genotyped 95 of the isolates collected in this study with nine previously developed microsatellite markers (57). We found high genetic diversity among isolates collected from the same settings and substrates and isolates from different settings and substrates, indicating that populations were not structured based on the product of origin, environment, or geographic location (Fig. 3 and 5). We did find that isolates with TR alleles and pan-azole resistance were genetically structured into a distinct cluster or lineage, which is consistent with previous studies (31, 33, 66–68). Many studies have compared pan-azole-resistant isolates from clinics and agricultural environments and found that these isolates cluster based on the *cyp51A* allele rather than the environment of origin (5, 8, 66, 83). These analyses have found that there are usually two or three primary clusters or clades structured by pan-azole resistance and the presence of a tandem repeat in the promoter or lack thereof (31, 33, 66–68). With DAPC (65), we identified six genetic clusters among our isolates from retail products in the U.S. and environmental and clinical isolates collected from Georgia and Florida. Isolates from Clusters 1 and 6 belonged to distinct branches in the minimum spanning networks based on both Nei’s genetic distance (62) and Bruvo’s genetic distance models (60), supporting genetic structure among isolates in those branches and the rest of the isolates (Fig. 5). Isolates within these two clusters had similar *cyp51A* genotypes, with the majority of isolates having TR alleles in Cluster 6 and the F46Y/M172V/E427K allele in Cluster 1, which suggests that the clusters are somewhat structured by both *cyp51A* genotype and azole resistance phenotype (Fig. 5). An isolate from retail soil was an exception, where it had the TR_46_ allele but belonged to Cluster 5. This isolate could be a recombinant since it shares all nine STR*Af* alleles with an isolate from the same sample; however, that isolate is azole susceptible and was not sequenced at *cyp51A*. The isolates found in Cluster 1 were exclusively collected from retail products in this study and all had the F46Y/M172V/E427K allele. A cluster of isolates only from commercial products has not been reported in previous studies. More research is needed to determine if this cluster is more widely present in other environments and if these isolates cluster due to a reason other than retail product origin or their *cyp51A* genotype. Another explanation for this could be that these alleles could have arisen through microsatellite homoplasy, which may misrepresent haplotype distance using the stepwise mutation model (84).

More research is required to determine how widespread azole resistance is in the environment, find new locations and reservoirs of these isolates, and identify hotspots where azole resistance is prevalent. Surveillance of additional environments and substrates for azole-resistant *A. fumigatus* is needed, especially in the U.S., where environments have not been investigated as thoroughly as in Western Europe, India, and other regions around the world. Consistent with previous studies (3, 27, 35, 39), we found that flower bulbs and compost were hotspots of azole-resistant *A. fumigatus*; however, we identified that retail bulbs and compost are reservoirs and potential drivers of long-distance dispersal of antifungal-resistant isolates. Moreover, we identified that retail raw peanuts, almonds, and pecans are potential reservoirs of fungicide-resistant *A. fumigatus*. These food items and hotspot landscape products could pose a risk to people who are immunocompromised or have decreased lung function, so we suggest caution for at-risk groups when interacting with these products. Determining the population structure and diversity of clinical and environmental *A. fumigatus* isolates is important to assess risk for the development of azole resistance in different environments and to elucidate the evolutionary origins of these mechanisms of azole resistance in the environments. Identifying the evolutionary relationship among the isolates in this study and other studies could help point to how these genotypes are arising and spreading around the globe and could eventually assist in preventing azole-resistant aspergillosis infections in susceptible patients in high-risk environments.

## Data Availability

Isolates from this study will be made available upon reasonable request. All *cyp51A* sequence data and STR*Af* genotype data are available on Dryad at 10.5061/dryad.zs7h44jfd.

## SUPPLEMENTAL MATERIAL

Table S1 aem.02056-23-s0001.xlsx Samples of *Aspergillus fumigatus* collected from retail products.

ASM does not own the copyrights to Supplemental Material that may be linked to, or accessed through, an article. The authors have granted ASM a non-exclusive, world-wide license to publish the Supplemental Material files. Please contact the corresponding author directly for reuse.
